# Tooth loss during long-term periodontal therapy in specialized practices – a retrospective cohort study from a periodontal practice-based research network (Perio-PBRN)

**DOI:** 10.1007/s00784-024-05993-9

**Published:** 2024-10-23

**Authors:** Steffen Rieger, Helena Walker, Felix Mittelhamm, Eberhard Frisch, Stefanie A. Peikert, Anne B. Kruse, Nils B. Liedtke, Petra Ratka-Krueger, Kirstin Vach, Johan P. Woelber

**Affiliations:** 1 Department of Dental Medicine and Oral Health, Medical University of Graz, Billrothgasse 4, Graz, 8010 Austria; 2 Department of Operative Dentistry and Periodontology, Faculty of Medicine, University of Freiburg, Hugstetter Straße 55, 79106 Freiburg, Germany; 3 Private Dental Practice, Reutlingen, Germany; 4 Private Dental Practice, Malters, Switzerland; 5 Private Dental Practice, Hamburg, Germany; 6 Private Dental Practice, Hofgeismar, Germany; 7 Department of Odontology, University of Copenhagen, Nørre Allé 20, Copenhagen N, 2200 Denmark; 8 Hannover Medical School (MHH), Department of Conservative Dentistry, Periodontology and Preventive Dentistry, Carl-Neuberg-Str. 1, 30625 Hannover, Germany; 9 Policlinic of Operative Dentistry, Periodontology, and Pediatric Dentistry, Medical Faculty Carl Gustav Carus, Technische Universität Dresden, Fetscherstraße 74, 01307 Dresden, Germany; 10 Institute of Medical Biometry and Statistics, Medical Center - University of Freiburg, Stefan-Meier-Str. 26, 79104 Freiburg, Germany

**Keywords:** Tooth loss, Periodontitis, Periodontics, Retrospective study, Practice-based research

## Abstract

**Objectives:**

To investigate tooth-specific, clinical tooth-, and patient-related factors associated with tooth loss (TL) in patients with mild to severe periodontitis treated in a periodontal practice-based research network (Perio-PBRN) over at least 5 years.

**Materials and methods:**

The Perio-PBRN consists of five German periodontal practices where clinical data were collected regarding patient age, tooth type, bleeding on probing (BOP), pocket probing depth (PPD), furcation involvement (FI), number of attended appointments, and other variables of interest. The data were evaluated regarding factors influencing TL.

**Results:**

Data from 687 patients (aged 54.5 ± 11.1 years) with mild (*N* = 23, 3.35%), moderate (*N* = 247, 35.95%) or severe (*N* = 417, 60.70%) periodontitis and 15,931 teeth over a mean observation period of 6.83 ± 1.40 years per patient were gathered via the Perio-PBRN. In this period, a total of 657 teeth were lost (4.12%, overall TL: 0.14 ± 0.22 teeth/patient/y). The risk of TL was significantly increased for teeth with persistent PPD ≥ 6 mm (hazard ratio: 6.81 [95% confidence interval: 5.07–9.15] in comparison to PPD < 4 mm. Additionally, BOP (3.90 [2.46–6.19]), furcation involvement, jaw, age and tooth type showed a significant influence on TL, while number of visits were not significantly associated with TL.

**Conclusions:**

Periodontal care of patients with moderate to severe disease in specialized practices was associated with a low rate of TL. Known tooth-related prognostic factors were confirmed. However, the results must be interpreted cautiously without knowledge of risk factors such as smoking and diabetes.

**Clinical relevance:**

PBRNs can help gather large amounts of periodontal practice-level data.

**Trial registration:**

The study was registered in the German Clinical Trials Register (DRKS 00011448).

## Introduction

Periodontitis is characterized by tissue destruction of the periodontium, which can lead to progressive attachment loss and eventually tooth loss [1]. Accordingly, the main goal of periodontal therapy is to preserve the teeth and surrounding periodontal tissues in a healthy, painless, aesthetically and functionally good condition. To evaluate the efficiency of systematic periodontal therapy, frequently used surrogate parameters such as bleeding on probing (BOP), pocket probing depth (PPD), or clinical attachment loss (CAL) have been shown to be sensitive and sufficient risk parameters for the stability or progression of the disease [2]. As a true final parameter, however, it has been proposed that tooth loss (TL) be used as the primary outcome parameter in studies [3]. This parameter is reliable to measure and is a severe event for the patient, accompanied by a significant reduction in the quality of life [4] and usually accompanied by complex and expensive treatments [5].

Several studies have addressed tooth-related, site-related and patient-related risk factors for TL [6–8]. In a recent review and meta-analysis, Helal et al. (2019) investigated the association between TL and several risk factors in periodontitis patients. Their principal findings were that older age, nonadherent to treatment, smoking, diabetes, and teeth with advanced bone loss, high PPD, tooth mobility, or molars, especially with furcation involvement, were associated with higher risks of TL [6].

This systematic review also reflected an imbalance between data from university settings and data from practice settings. Only five of the 20 studies included had been performed in practice, whereas one of these studies analysed only patients’ records from different general dental practices without including detailed information about the periodontal examination [9]. The other studies performed in practice only had a small sample size, a total of 474 patients. Accordingly, even well-conducted systematic reviews are currently not able to gather enough patient data about periodontal therapy on a practice level. From the perspective of health care research, the discrepancy in terms of research data between university and practice settings is critical since everyday treatment modalities might considerably differ from research settings at universities [10, 11].

One approach to overcome this imbalance is the use of patient data from practice-based research networks (PBRNs), which offers the great advantage of obtaining data from many practices under everyday conditions [12]. The connection of dental practices to PBRNs is strongly supported by the ongoing digitization of health care [13], which allows us to gather and synthesize vast amounts of clinical data. These data can be used for clinical decision support, disease surveillance and population health management [14].

In a survey of Swiss dentists, the relative odds of not extracting teeth with severe periodontitis were higher if the dentist was a periodontal specialist rather than a generalist [15]. Consequently, it seems useful to distinguish the practice level between general practices and specialized periodontal practices in addition to the university setting. Interestingly, all practice based studies conducted between 1978 [16] and 2017 [17], which followed patients regarding TL and other parameters, were performed by specialist practices [18]. Classic studies have already shown that with treatment and periodic maintenance care progress of periodontitis is minimal, and TL is rare – with TL rates between 0.2 and 3.6, i.e. number of teeth lost per patient over length of the study [19].

A recently established periodontal practice-based research network (Perio-PBRN) showed in a proof-of-concept, that it was possible to collect anonymized digital periodontal data from 6,301 patients from nine specialized periodontal practices and thus include sufficiently large amounts of data for evaluation in health care research studies [20]. To the best of the authors’ knowledge, there are no comparable data from a specialized periodontal practice level available regarding TL during periodontal therapy.

Thus, the objective of this retrospective study was to assess TL in periodontitis patients treated in a Perio-PBRN and to identify tooth-specific, clinical tooth-, and patient-related factors influencing long-term TL during active and supportive periodontal therapy.

## Materials and methods

### Patients and Perio-PBRN data analysis

The periodontal patient data were based on a German periodontal practice-based research network (Perio-PBRN) [20]. This study was reviewed and approved by the University of Freiburg Ethics Committee (EK 493/16), internationally registered in the German Clinical Trials Register (DRKS 00011448) and conducted in accordance with the Helsinki Declaration of 1975, as revised in 2013.

The Perio-PBRN consisted of nine practices, whose dentists were all alumni of the master’s programme in periodontology and implant therapy of the University of Freiburg, Germany [21]. Due to their similar postgraduate training, the participating dentists had been taught a standard stepwise treatment protocol according to approved guidelines [22, 23]. This ensured a similar level of education and a basic calibration of the participants, while treatment modalities could differ due to practitioner-individual treatment preferences. Each participant was informed about the structure and the study design of this trial and signed a consent form.

All practices had to routinely use a digital periodontal examination programme (ParoStatus^®^, ParoStatus.de GmbH, Berlin, Germany) for documentation purposes in their practice and to transfer the anonymized patient data. This software is widely used in Germany, accredited by the standards of the German Periodontal Society and includes the monitoring of all relevant periodontal treatment parameters [20], as described below. It can be easily integrated in the daily routine. All transferred patient data had to meet the following inclusion criteria: (i) confirmed diagnosis of periodontitis according to the CDC-AAP definitions for population-based studies [24]; (ii) data collection period of at least 5 years; and (iii) at least 3 visits to the practice. The CDC-AAP definitions are characterized as follows [24]: No periodontitis (no evidence of mild, moderate, or severe periodontitis); mild periodontitis (≥ 2 interproximal sites with CAL ≥ 3 mm, and ≥ 2 interproximal sites with PPD ≥ 4 mm (not on same tooth) or one site with PPD ≥ 5 mm); moderate periodontitis (≥ 2 interproximal sites with CAL ≥ 4 mm (not on same tooth), or ≥ 2 interproximal sites with PPD ≥ 5 mm (not on same tooth)), and severe periodontitis (≥ 2 interproximal sites with CAL ≥ 6 mm (not on same tooth) and ≥ 1 interproximal site with PPD ≥ 5 mm). A visit was defined as such when a periodontal examination had been recorded. Visit 1 represents the baseline evaluation. Between visits 1 and 2, active periodontal therapy (APT) was performed, while the following visits were considered to belong to the supportive periodontal care (SPC) phase. All practices used similar SPC-regimes based on their calibrated education including risk-orientated intervals [25]. However, this phase lacked information regarding possible additional therapies (e.g., periodontal surgery).

The transferred clinical data included the following parameters:

Tooth-specific factors:

- Tooth type differentiated into incisor, canine, premolar, and molar.
- Upper or lower jaw.
- Furcation involvement (FI) assessed by 4 degrees: 0, I, II, and III [26] using a Nabers probe.
- Tooth mobility (M) assessed by 4 degrees: 0, 1, 2, and 3 [27, 28].

Clinical tooth-related factors (assessed with the University of North Carolina (UNC) 15 probe), each of them measured at 6 sites per tooth:

- Pocket probing depth (PPD) in mm.
- Bleeding on probing (BOP) as presence/absence.
- Gingival recession (R) in mm.
- Clinical attachment loss (CAL) in mm.

Patient-related factors:

- Patient age in years at baseline.
- Visits by observation time. Therefore, the quotient of the total number of visits and the total observation time in years was calculated. The more often the patient visited the dentist, the greater the index.

### Statistical procedures

#### Definition of outcome measures

In this study different outcomes were of interest. We distinguished between variables at the patient level and tooth level. In addition to the factors that could be clearly assigned to the tooth-level independent of time, such as jaw and tooth type, we used the mean value of the BOP, R or PPD over the entire period per tooth as an indication of dental health during the observation period. That is, it was averaged over the 6 measuring points per tooth and the time the tooth was under observation. This measure for BOP gave an indication of whether the bleeding was very infrequent or a constant problem. PPD, R and M were measures of tooth behavior over the entire period. For some of the clinical parameters (FI, M) the corresponding value at baseline was considered to make a statement about the prognosis. For BOP and PPD the corresponding value after APT was considered as indicator for the success of therapy.

For the graphical presentations some outcomes were categorized. In detail, the following outcome measures were used:

BOP:

- Tooth time average (relative frequency of bleeding measured at 6 points over the whole observation time).
- Additionally, BOP after APT was classified into three categories (0 or 1, 2 or 3, and 4 or more out of 6 measuring points show bleeding).

PPD:

- Tooth time average (mean over the whole observation time).
- Tooth average, classified into three categories (< 4 mm/4, 5 mm/≥ 6 mm) [23].
- Mean value per tooth after APT.
- Maximal value per tooth at baseline.

Combining PPD and BOP:

- Building 6 groups by combining maximal value per tooth after APT classified into three categories (< 4 mm/4, 5 mm/≥ 6 mm) with mean of bleeding on probing after APT over the whole observation time ≤ 10% versus > 10%.

FI:

- Furcation involvement per tooth at baseline categorized as FI-0, FI-I, FI-II, and FI-III.
- Furcation involvement per tooth after APT categorized as FI-0, FI-I, FI-II, and FI-III.

M:

- Tooth-specific value at baseline with degrees 0 to 3. As M is difficult to assess reproducibly, we combined degrees 0 and 1.
- Tooth-specific value after APT with degrees 0 to 3. As M is difficult to assess reproducibly, we combined degrees 0 and 1.

R & CAL:

- Tooth time average (mean over the whole observation time).

Medians, mean values, and standard deviations were calculated for a descriptive analysis of the data. Kaplan‒Meier (KM) survival rates, including 95% confidence intervals, were calculated. Cox regression models (with consideration of the clustering of the teeth in the case of analyses at the tooth level) were used for group comparisons. KM-curves were used for a graphical representation.

In the analyses, the results are presented at different points in time. As mentioned above, between the 1st and 2nd visits, APT took place. Since the patients had been in treatment for at least 5 years, but for very different lengths of time, the results for the last visit (possibly the deadline for data collection or the cessation of treatment) are also presented. Since the annual frequency of visits also varied both between and within patients, year-based analyses are given. All analyses were performed with the statistical software STATA 17.0 (StataCorp LLC, Lakeway Drive College Station, Texas, USA). *p* < 0.05 was considered as statistically significant. All used regression models accounted for clustering of the data.

## Results

### Baseline data and overview of TL

A total of 687 patients (mean age at baseline: 54.5 ± 11.1 years) with 15,931 teeth from five practices over a mean observation period of 6.83 (± 1.40) years fulfilled all inclusion criteria. At baseline, the number of teeth was significantly lower with increasing patient age (*p* < 0.001, Table 1). CDC-AAP definitions revealed that 664 (96.65%) patients were diagnosed with moderate or severe periodontitis (Table 2).

**Table 1:** Tooth/age distribution at baseline (without third molars)

Patient age (years)	*N*	Mean (± SD) *N* tooth baseline	Median
< 50	263	24.87 ± 3.38	26
50–60	215	22.64 ± 5.13	24
60–70	140	22.46 ± 5.02	24
> 70	69	19.97 ± 5.75	21
Total	687	23.19 ± 4.82	24

**Table 2:** Patient-specific disease severity

Case definition ^a^	Baseline *N* (%) patients	After APT *N* (%) patients	Last visit *N* (%) patients
No periodontitis		26 (3.78)	52 (7.57)
Mild periodontitis	23 (3.35)	22 (3.20)	15 (2.18)
Moderate periodontitis	247 (35.95)	306 (44.54)	360 (52.40)
Severe periodontitis	417 (60.70)	333 (48.47)	260 (37.85)

^a^ according to the CDC-AAP case definitions [24]

**Fig. 1:**
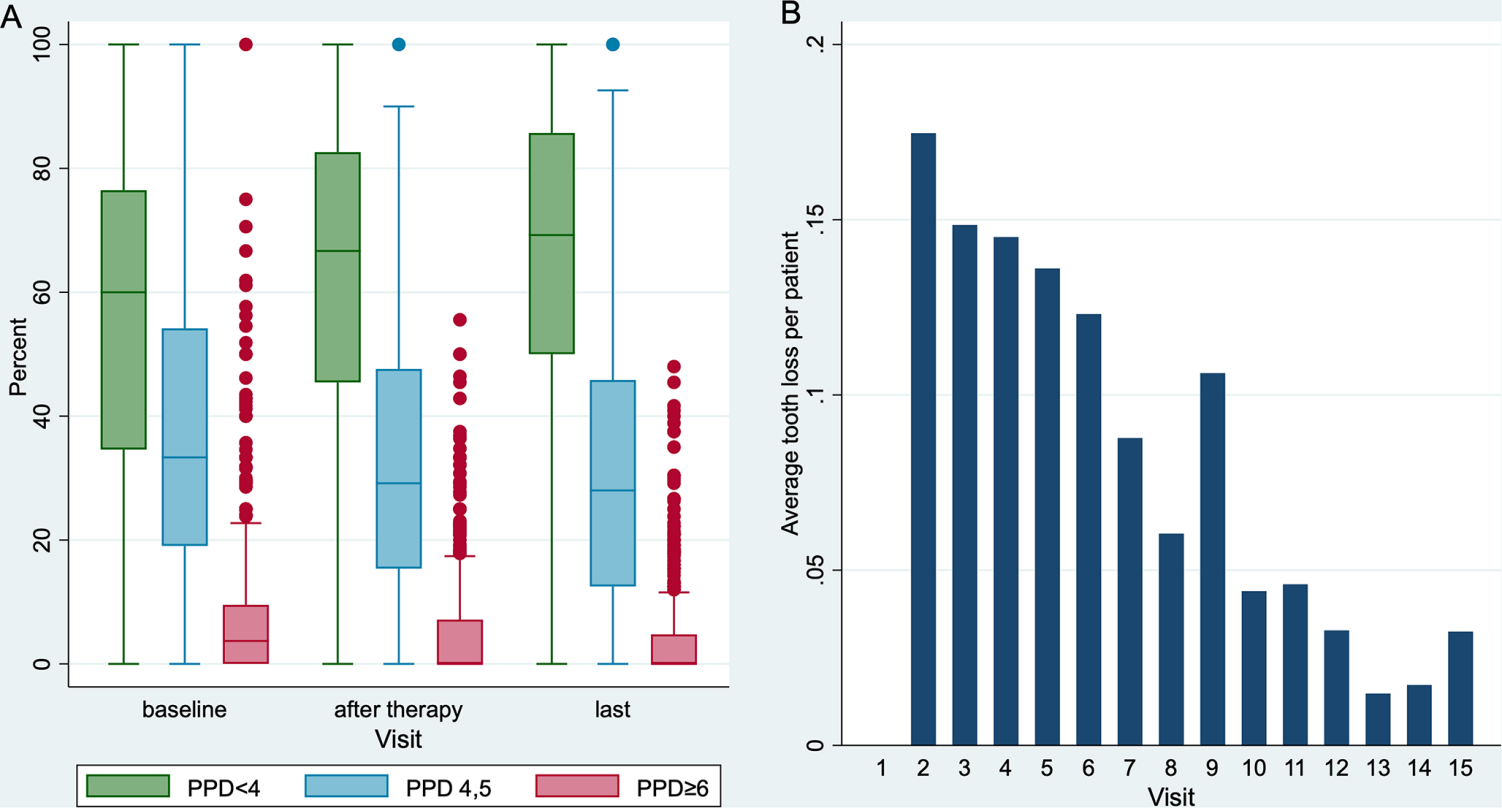
(**A**) Proportion of teeth by PPD at baseline, after APT and the last visit and (**B**) average tooth loss per patient per visit

When stratified into three categories (< 4 mm/4, 5 mm/$\:\ge\:$ 6 mm), the proportion of teeth with PPD < 4 mm increased from 55.8 to 63.0%, while the proportion of teeth with PPD $\:\ge\:\:$6 mm decreased from 7.3 to 4.7% from before to after therapy (Fig. 1A). The time between baseline and after APT was 0.7 years on average. At the last visit, the proportion of teeth$\:\:\ge\:\:$6 mm was even lower (3.9%). If we define teeth requiring treatment as those with PPD $\:\ge\:$ 5 mm or PPD of 4 mm with simultaneous BOP, the number of teeth requiring treatment at baseline was reduced from 2,457 (15.3%) to 1,394 by APT. A stable condition was thus achieved in 43.3% of the teeth.

Of the 687 included patients, 383 patients did not lose a tooth (55.75%), 150 patients lost 1 tooth (21.83%), 63 patients lost 2 teeth (9.17%), 39 patients lost 3 teeth (5.68%), and 52 patients lost 4 or more teeth (7.57%). In sum, a total of 657 teeth were lost (4.12% of 15,931 teeth).

120 (18.26%) of all total lost teeth were extracted between baseline and after APT. TL events decreased over the course of treatment (Fig. 1B). Over the entire observation period of 6.83 years per patient (SD: 1.40, median: 6.94), this corresponded to a TL of 0.96 teeth/patient and a mean annual TL of 0.14 ± 0.22 teeth/patient.

### Tooth-specific factors for TL

More than half of the lost teeth were molars (45.21%; 297 teeth), followed by premolars (29.68%; 195 teeth) and incisors (17.96%; 118 teeth). Canines were lost the least frequently, making up 7.15% (47 teeth). The results of Cox regression analysis (with the reference canine) showed a 1.31-fold increased risk of TL for incisors. Premolars (HR = 2.20) and molars (HR = 3.39) also showed a higher risk (Table 3). Eight-year KM survival probabilities per tooth type were 98.3% for canines, 97.6% for incisors, 96.3% for premolars, and 93.8% for molars.

Looking at the TL differentiated by jaw, a slightly higher prevalence for TL in maxillary teeth (59.51%; 391 teeth) compared to mandibular teeth (40.49%; 266 teeth) was found. Cox regression analysis showed that the risk for TL was significantly lower in the mandible than in the maxilla (HR = 1.50, *p* < 0.001, Table 3). Eight-year KM survival probabilities were 95.6% for the maxilla and 96.7% for the mandible.

**Fig. 2:**
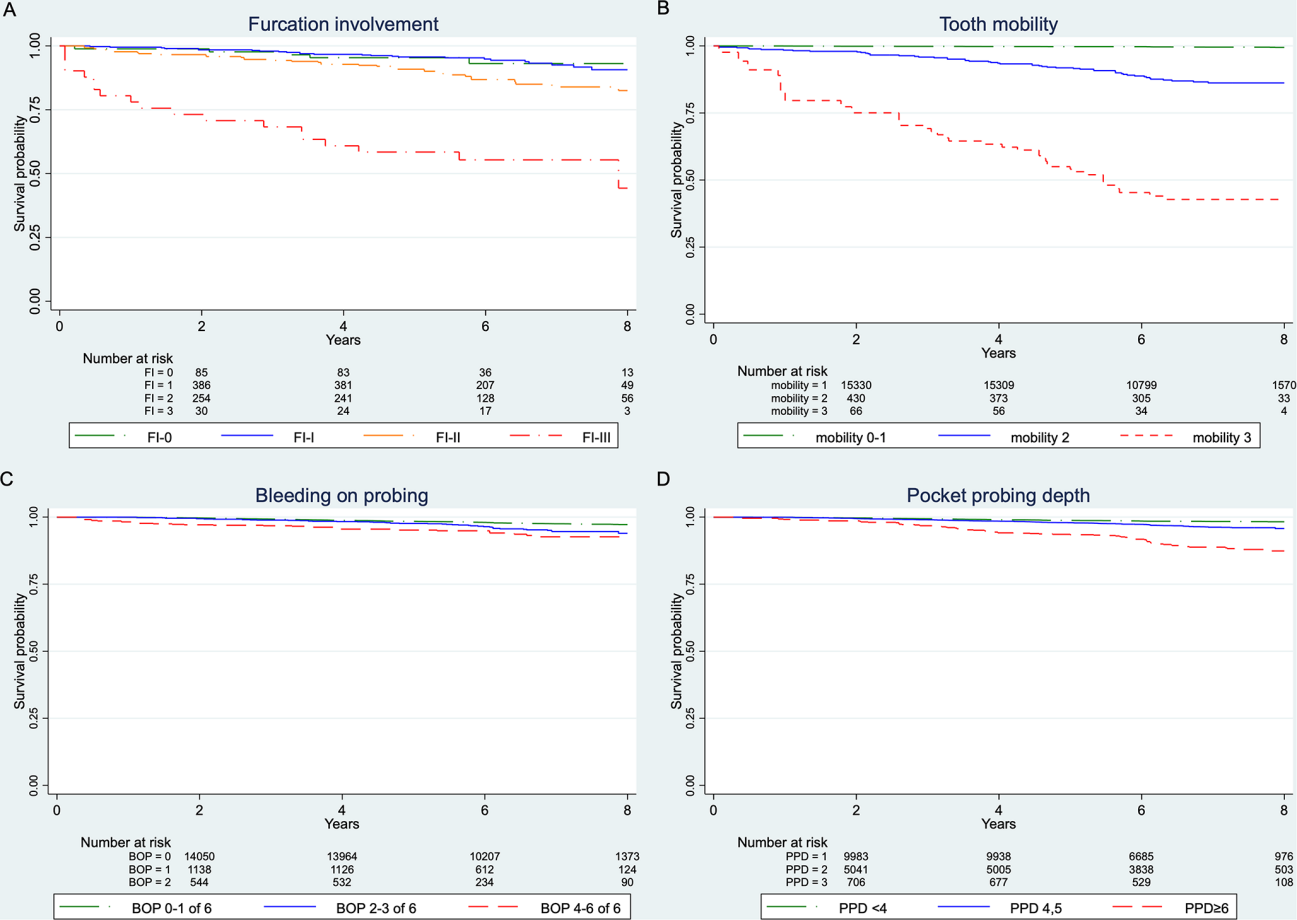
Kaplan‒Meier survival probability of teeth according to (**A**) furcation involvement at baseline, (**B**) tooth mobility at baseline, (**C**) tooth BOP after APT in categories and (**D**) PPD after APT in categories

Cox regression analysis showed a clear association between FI and TL. The pairwise comparison to FI-0 at baseline showed a 1.2-fold increased risk for FI-I, a 2.4-fold increased risk for FI-II and a 10.3-fold increased risk for FI-III (Table 3). However, due to the small number of cases for FI-III, the results should be viewed with caution (Fig. 2A).

Eight-year KM survival probabilities were 93.1% for FI-0, 90.7% for FI-I, 82.6% for FI-II, and 44.3% for FI-III (Fig. 2A). When considering FI after APT the HR were even larger in comparison to baseline.

Regarding M at baseline, 1.07% (171 of 15,931 teeth) of mobile teeth (degrees 1 to 3) were lost. The risk of losing a tooth was increased by a factor of 7.8 when M was present at baseline at level 2 or higher (*p* < 0.0001) and was still increased by a factor of 6.1 if M was at level 2 or higher after therapy (*p* < 0.001). Five-year KM survival probabilities for M at baseline were 99.7% for M-0-1, 91.8% for M-2, and 54.0% for M-3 (Fig. 2B), while the eight-year KM survival probabilities were not calculated because of too little patient data.

### Clinical tooth-related factors for TL

Cox regression analysis based on the averaged BOP value of tooth sites over the observation period revealed that TL was significantly dependent on BOP. The HR of TL was 4.66 (*p* = 0.001, Table 3). When the tooth’s BOP value after APT, which shows an HR of 3.90, was differentiated into three categories (0–1/6, 2–3/6, 4–6/6), the eight-year KM survival probabilities were 97.2% for < 0–1, 94.0% for 2–3 and 92.6% for 4–6 (Fig. 2C).

Per each mm increase in the mean PPD value of a tooth over the observation period, the risk of TL was increased by a factor of 1.40 (*p* < 0.0001, Table 3). This value increased to 1.44 if we considered the maximal PPD per tooth at baseline and to 1.48 if we considered the mean PPD value after APT. If this PPD value was differentiated into three categories (< 4 mm/4, 5 mm/≥ 6 mm), the eight-year KM survival probabilities were 98.3% for < 4 mm, 95.8% for 4, 5 mm and 87.4% for ≥ 6 mm (Fig. 2D). Compared to < 4 mm, the risk of TL increased by a factor of 2.17 for 4, 5 mm (95% CI [1.72–2.73]) and by a factor of 6.81 for ≥ 6 mm (95% CI [5.07–9.15]). Both hazard ratios were significant (*p* < 0.001).

**Fig. 3:**
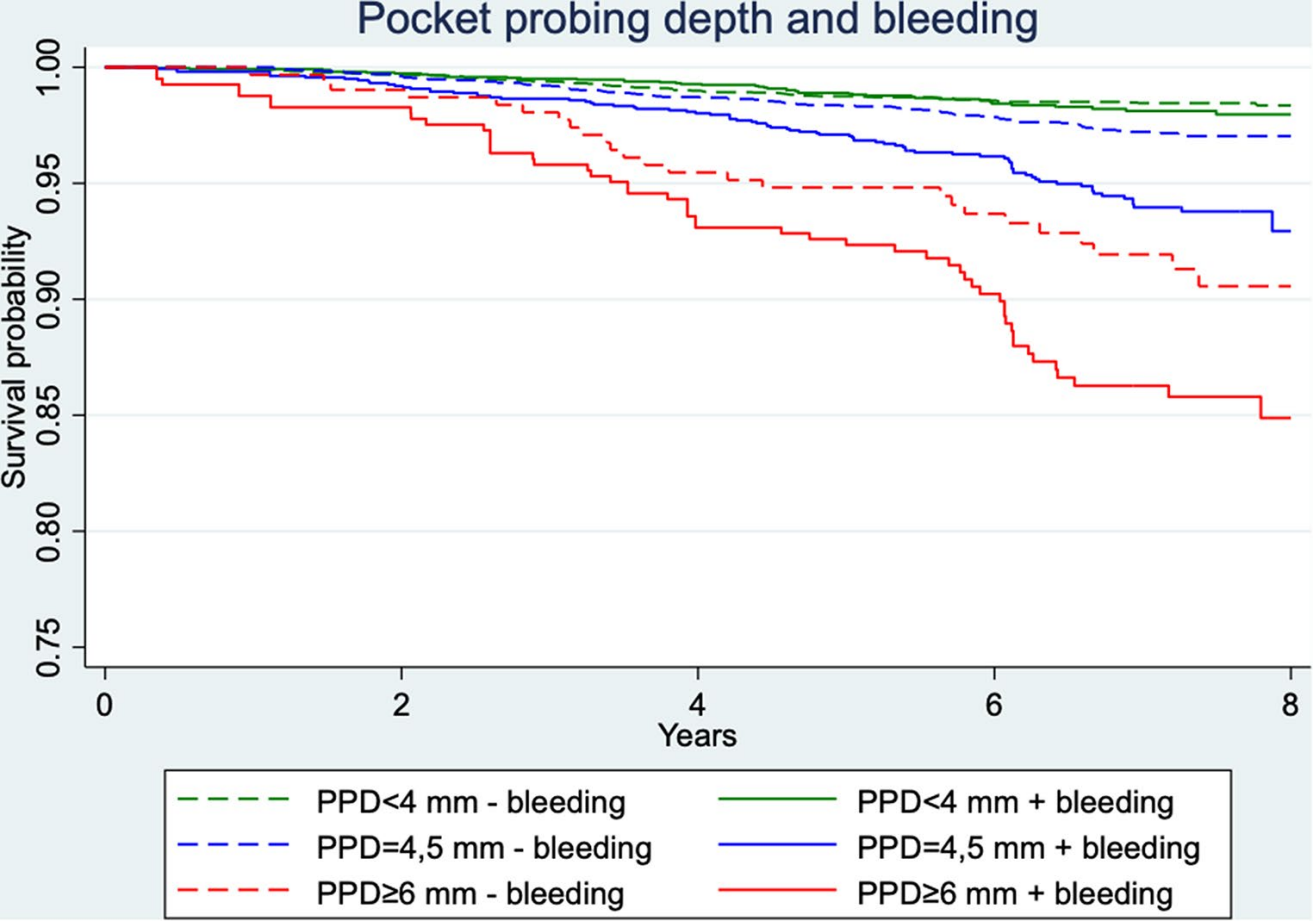
Kaplan‒Meier survival probability of teeth according to maximal PPD per tooth after APT in categories < 4 mm, 4, 5 mm and ≥ 6 mm combined with mean value of bleeding on probing after therapy over the whole observation time ≤ 10% (- bleeding) or *>* 10% (+ bleeding)

When combining the categorized (< 4 mm/4, 5 mm/≥ 6 mm) maximal PPD values after APT with the proportion of BOP measured after APT over the whole observation time categorized in ≤ 10% and > 10%, we observed more TL (Fig. 3) for all 3 PPD categories if bleeding was present. Results of a cox regression analysis comparing bleeding ≤ 10% and > 10% showed a HR of 1.19/1.89/1.46 for the PPD categories < 4 mm/4, 5 mm/≥ 6 mm with a significant result (*p* = 0.008) for the 4, 5 mm group.

Mean CAL was also significantly associated with TL, while mean R did not reach significance at a 95% level (Table 3).

### Patient-related factors for TL

The risk of TL within the 5-year observation period depended on the patient’s age (HR = 1.01; *p* = 0.006, Table 3).

In the Cox regression model regarding visits by observation time, the HR was 1.20 while not reaching significance at a 95% level (*p* = 0.055, Table 3). Thus, patients with more frequent visits showed a trend towards a higher rate of TL.

### Analyses on practice-level

An annual TL of 0.14 ± 0.22 per patient was determined across all practices and patients over the entire observation period of 6.83 years. However, there was a significant difference in TL within the first 5 years observed across the practices (*p* = 0.0001). Apparently, practice 5 had a high impact on the results as it contributed the most patient data. In contrast, data from practice 4 should be viewed with caution, as this practice contributed few patients (Table 4).

**Table 3:** Results from Cox regression analyses (*N* = 15,931 teeth)

Variable	HR TL	95% CI	*p*-value
Tooth type Canine (ref.) Incisor Premolar Molar	1.00 1.31 2.20 3.39	0.86–1.98 1.46–3.29 2.27–5.07	< 0.0001
Jaw Mandible (ref.) Maxilla	1.00 1.50	1.22–1.85	< 0.001
Furcation involvement (FI) BL Degree 0 (ref.) Degree I Degree II Degree III	1.00 1.20 2.38 10.31	0.40–3.58 0.77–7.38 3.08–34.51	< 0.0001
Furcation involvement (FI) APT Degree 0 (ref.) Degree I Degree II Degree III	1.00 1.50 4.83 15.41	0.48–4.63 1.51–15.39 4.77–49.86	< 0.0001
BOP (tooth time average)	4.66	1.85–11.71	0.001
BOP (after APT)	3.90	2.46–6.19	< 0.001
PPD (tooth time average) [mm]	1.40	1.26–1.55	< 0.001
PPD (after APT) [mm]	1.48	1.38–1.58	< 0.001
Gingival recession (R) [mm]	1.14	0.99–1.30	0.066
CAL [mm]	1.31	1.17–1.46	< 0.001
Age [years]	1.01	1.00-1.02	0.006
Visits by observation time	1.20	0.996–1.44	0.055

HR, hazard ratio; TL, tooth loss; CI, confidence interval; ref.: reference; BOP, bleeding on probing as relative frequency; PPD, pocket probing depth; CAL, clinical attachment loss; BL, baseline

**Table 4:** Analyses on practice-level

Practice	*N* patient data	Mean observation time per patient in years (± SD)	Practice maximal observation time in years	Mean *N* tooth baseline (± SD)	*N* teeth lost first 5 years	Mean annual TL first 5 years (± SD)
1	17	5.18 (± 0.13)	5.58	22.88 (± 4.93)	7	0.08 (± 0.15)
2	69	6.32 (± 0.97)	8.87	22.07 (± 5.94)	33	0.08 (± 0.16)
3	52	7.67 (± 2.10)	13.09	25.61 (± 2.34)	49	0.13 (± 0.20)
4	17	6.72 (± 1.57)	9.45	23.71 (± 3.24)	33	0.27 (± 0.42)
5	532	6.87 (± 1.31)	17.63	23.09 (± 4.82)	535	0.15 (± 0.22)
Total	**687**	**6.83** (± 1.40)	**17.63**	**23.19** (± 4.19)	**657**	**0.14** (± 0.22)

TL, tooth loss

## Discussion

The present study retrospectively investigated TL over at least 5 years in the context of tooth-specific, clinical tooth-, and patient-related risk factors in a large periodontitis patient population of a periodontal practice-based research network (Perio-PBRN). TL was significantly correlated with tooth type, jaw, FI, M, BOP, PPD, CAL, age and the related practice. The risk factors PPD ≥ 6 mm (compared to < 4 mm), M degree 2 and higher (compared to degree 0 or 1) and FI-III (compared to degree 0) thereby showed the strongest association with TL.

4.12% of 15,931 investigated teeth were lost overall, which corresponded to an average TL of 0.96 teeth/patient and a mean annual TL of 0.14 per patient. In their systematic review, mainly based on data from university centres, Helal et al. found a mean annual TL/patient of 0.12 (min/max: 0.01/0.36) [6]. This is a slightly lower than in the present study, which represents data exclusively from specialized periodontal practices. Compared to other specialized practice-based studies, we found similar results regarding TL. Two studies are cited as examples: In their classic study, Hirschfeld & Wassermann determined an average tooth loss of 1.8 per patient (average observation time after APT: 22 years, 600 patients) [16]. In their more recent study, Nibali et al. determined an average tooth loss of 1.1 per patient with an average observation period similar to ours (6.6 years after APT) and 100 patients [17].

Comparing the current data from the Perio-PBRN with data from German general dental practices, the TL rate of the present study was much lower: Raedel et al. extracted data from the digital database of a major German national health insurance company (Barmer) and could trace a total of 415,718 periodontal treatments. The cumulative four-year tooth survival rate was 63.8% after periodontal treatment [29], while this rate was above 95% in the present study. However, Raedel’s data referred to patients covered under statutory health insurance only. The insurance status was not known for the current Perio-PBRN data. However, it can be assumed that the current data were based on a similar proportion of approximately 87% of patients with statutory insurance, as found in other German population studies [18]. Patients under a private insurance plan may have a lesser probability of TL as shown in other studies [30]. Accordingly, insurance status might have an influence on TL in the current data. Both specialists and generalists must follow statutory health insurance guidelines, which makes the data comparable to a certain extent. Kocher et al. recently determined the annual TL from a subset of the mentioned Barmer data to be 0.35 and related this to a control group of untreated patients (annual TL 0.19).

Interestingly, it was found that most teeth in the Barmer group were removed during an early stage of therapy [31]. This notable finding was also identified in the present study: 120 teeth (18.26%) were lost during APT, and the rate of loss decreased steadily during treatment. This implies not spontaneous tooth loss but a rational decision by the dentist that must be viewed rather critically at this early stage of therapy [32]. In the present, comparatively severely periodontally compromised [33, 34] patient population, no statement can be made regarding necessary prosthetic restorations to justify earlier extraction. However, the early loss of teeth with FI-III suggests a deliberate early extraction due to the limitations of treating molars with FI-III (in combination with degree 3 mobility) under the regulations of the statutory health insurance system in Germany [29] or in cases of pending prosthetic restoration.

Teeth with furcation involvement generally showed a significantly higher risk of TL in the present study, confirming the results of previous studies [35, 36]. Furthermore, the risk of TL increased according to the degree of furcation involvement. The values for FI-I and FI-II were similar to other recent study results [35]; however, the present study showed a noticeably higher HR for FI-III. As mentioned above, prosthetic, other nonperiodontal reasons or statutory regulations may also have supported a decision for extraction. Some tooth types appeared to be more susceptible to TL than others. Molars showed the highest risk of being lost ahead of premolars and incisors. In turn, canines showed the best prognosis. These observations in the distribution pattern of TL are similar to the results of previous studies [16]. The higher loss probability of molars was confirmed in many studies [37–39]. Reasons for this may be their poor accessibility for oral hygiene measures, especially in the furcation area [40]. In addition, molars are more frequently treated restoratively and/or endodontically, which may lead to TL due to nonperiodontal reasons [6].

Tooth mobility was also significantly associated with TL. Various earlier studies were able to show a similar correlation: The greater the mobility, the higher the risk of TL [41–43]. However, the reliability of determining the exact degree of mobility is limited [6]. Thus, we pooled mobility degrees 0 and 1 to reduce possible bias. Splinted teeth may cause bias as well [38, 44], as our dataset did not contain information about the presence of periodontal splinting.

BOP serves as an important parameter for monitoring periodontal disease [45]. In the present study, the tooth time average BOP value was strongly associated with TL. A persistently high value of BOP-positive sites indicated pronounced inflammation of the gums and ongoing gingival inflammation is acknowledged as risk factor for TL [46].

Increased PPDs were significantly associated with a higher risk of TL, with an increased hazard of 1.40 per mm PPD, similar to other longitudinal studies [2, 36, 41]. Compared to PPD < 4 mm, the risk of TL increased by a factor of 6.81 for PPD ≥ 6 mm, thus PPD ≥ 6 mm represents inadequate periodontal treatment and should receive further therapy [39], which is also reflected by current guidelines [23]. On the other hand, after 5 years of therapy, more than 75% of teeth with persistent PPD ≥ 6 mm were still present.

The goal of therapy, however, is to obtain shallow PPD and absence of bleeding, indicating resolution of the inflammatory lesion [47]. Thus, we combined the categorized (< 4 mm/4, 5 mm/≥ 6 mm) maximal PPD values after APT with the proportion of BOP measured after APT over the whole observation time categorized in ≤ 10% and > 10% and observed more TL for all 3 PPD categories if bleeding was present, although only the 4, 5 mm group reached statistical significance. This is also shown in other studies: The presence of BOP in deep pockets indicates ongoing inflammation, which can lead to further periodontal deterioration and TL [2, 48]. On the other hand, this confirms that absence of BOP indicates periodontal stability [45]. Interestingly, the 10%-BOP-threshold for distinction between gingival health and disease is also confirmed indirectly by our data [49].

The number of teeth at baseline was significantly lower with increasing age, and patient age also showed a significant but rather weak effect on TL within the 5 years of observation in the present data. This result is consistent with various other studies [42, 50, 51]. However, some authors attribute limited relevance to age than to other, mainly tooth related, risk factors [36]. In a recent review, a significant but also rather weak association of patient age with TL was found, but only 8 of 20 included studies investigated this factor [6].

Patients with more visits per observation time showed a tendency towards a higher rate of TL. In this study, only the time points of visits at which a six-point attachment measurement was performed were registered, and not which SPC interval was actually scheduled. Hence, no statement can be made about patients’ adherence to treatment, which is an important patient-related factor affecting TL in the literature [7]. In a Swedish study on periodontal maintenance therapy 11–14 years after active periodontal treatment, the number of visits to dental hygienists was positively correlated with the number of lost teeth. The authors explained this with the assumption that patients with the most advanced periodontal disease were the most frequently called patients for maintenance treatments [52].

Regarding a possible centre effect, we analyzed various factors (Table 4). There was a significant difference in TL within the first 5 years observed across the practices. The assessment of tooth prognosis can be highly variable among different practitioners [53]. However, the baseline conditions for the practices were not the same; for example, the average age of the patients differed. Furthermore, the reasons that led to tooth extraction are unknown. It should also be noted that practice 5, with the most patients and a long observation period (Table 4), had the greatest impact on the results.

Retrospective cohort studies of periodontal PBRNs have several inherent strengths and limitations [20].

First, the investigated population must be critically discussed. Since only data from specialized periodontal practices were included, this also implies selection bias. Nevertheless, the data offered a view of a large population of periodontal patients who were treated in a practice setting. This is important since there is a lack of clinical studies from the daily routine of dentists in private practice compared to the extensive research in university institutions [11]. Most dentists are primarily engaged in private practice, and via a PBRN, practitioners can generate large amounts of “daily routine” clinical data that can be used to support evidence-based clinical practice [54]. However, large amounts of data must be interpreted carefully, as even smaller differences reach statistical significance. Therefore, the whole confidence interval and not only the p value should be considered, and emphasis should be placed on clinical relevance [55].

Second, it was not clear from the data how exactly active and supportive periodontal therapies were performed or whether there was prior periodontal treatment. However, all participating dentists were trained in the same manner with regard to periodontal assessment and therapy [21].

Third, due to limitations in the digital periodontal examination programme used and privacy considerations, there was a lack of further surrounding information. On the one hand, the program allowed the anonymized collection of periodontal data from many practices electronically without interfering with the daily routine. On the other hand, no information on reasons for tooth extraction, previous diseases and associated drug intake, sex, body mass index (BMI), oral hygiene and dietary habits, tobacco and alcohol consumption, physical activity, stress levels and vitamin D status was available to the study centre. These factors have an important influence on the severity of periodontal inflammation [56, 57]. Furthermore, retrospective staging and grading of periodontitis is not possible due to the lack of information on risk factors. This presents a major limitation of the current analyses. The naturally desirable recording of further patient-related parameters within the framework of health services research is, however, accompanied by restrictions at the level of data protection and ethics. Strict anonymization of patient data and secure data transfer from each practice must be ensured as a fundamental prerequisite.

In conclusion, the present study showed within its limitations that periodontal therapy of patients with moderate to severe disease in specialized practices was associated with a low annual TL of 0.14 ± 0.22 per patient on average. In absolute terms, 657 (4.12%) of 15,931 investigated teeth were lost over the average observation period of 6.83 years per patient. The recently established periodontal practice-based research network (Perio-PBRN) was able to collect large amounts of periodontal data. To improve data quality, it should be possible to collect more patient-related factors in the future if privacy and data protection can be ensured.

## Data Availability

No datasets were generated or analysed during the current study.
